# Mimics and Pitfalls of Primary Ovarian Malignancy Imaging

**DOI:** 10.3390/tomography8010009

**Published:** 2022-01-04

**Authors:** Sherif B. Elsherif, Ali Agely, Dheeraj R. Gopireddy, Dhakshinamoorthy Ganeshan, Karina E. Hew, Smita Sharma, Chandana Lall

**Affiliations:** 1 Department of Radiology, University of Florida College of Medicine—Jacksonville, Jacksonville, FL 32209, USA; DheerajReddy.Gopireddy@jax.ufl.edu (D.R.G.); Smita.Sharma@jax.ufl.edu (S.S.); Chandana.Lall@jax.ufl.edu (C.L.); 2 Department of Radiology, Mayo Clinic Florida, Jacksonville, FL 32224, USA; aliagely@gmail.com; 3 Department of Radiology, MD Anderson Cancer Center, Houston, TX 77030, USA; DGaneshan@mdanderson.org; 4 Department of Obstetrics and Gynecology, Division of Gynecologic Oncology, University of Florida College of Medicine—Jacksonville, Jacksonville, FL 32209, USA; Karina.Hew@jax.ufl.edu

**Keywords:** ovarian cancer, pelvic mass, adnexal mass, radiology interpretation

## Abstract

The complex anatomy and similarity of imaging features of various pathologies in the pelvis can make accurate radiology interpretation difficult. While prompt recognition of ovarian cancer remains essential, awareness of processes that mimic ovarian tumors can avoid potential misdiagnosis and unnecessary surgery. This article details the female pelvic anatomy and highlights relevant imaging features that mimic extra-ovarian tumors, to help the radiologists accurately build a differential diagnosis of a lesion occupying the adnexa.

## 1. Introduction

Adnexal masses have an incidence of 0.17% to 5.9% in asymptomatic women and 7.1% to 12% in symptomatic women, and less than 25% of these masses are diagnosed as malignancy [1]. The female pelvis and adnexa have complex anatomies. On imaging, ovarian and extra-ovarian processes occupying the adnexa can appear as an ovarian malignancy, which accounts for 4% of all cancers in women and is the leading cause of mortality from gynecologic malignancies. However, 5.1% of pelvic masses mimicking ovarian cancer are found to be an extra-ovarian disease [2]. Given that malignant and non-malignant processes share non-specific clinical symptoms, such as vague abdominal pain, abdominal and pelvic mass, weight loss, or vaginal hemorrhage, imaging plays a pivotal role in characterizing these lesions and assessing their origins [3]. Ultrasound is usually the primary modality in assessing a pelvic mass and MRI is the modality of choice for further characterization given its superior spatial resolution and soft tissue contrast [4]. To prevent unnecessary clinical and surgical interventions, the interpreting radiologist should be aware of imaging features that mimic ovarian cancer and can be mistaken for ovarian malignancy. This article will attempt to demystify the female pelvic anatomy and detail relevant clinical and imaging findings of several key extra-ovarian pathologies that can mimic ovarian malignancy.

## 2. Anatomy

**Figure 1:**
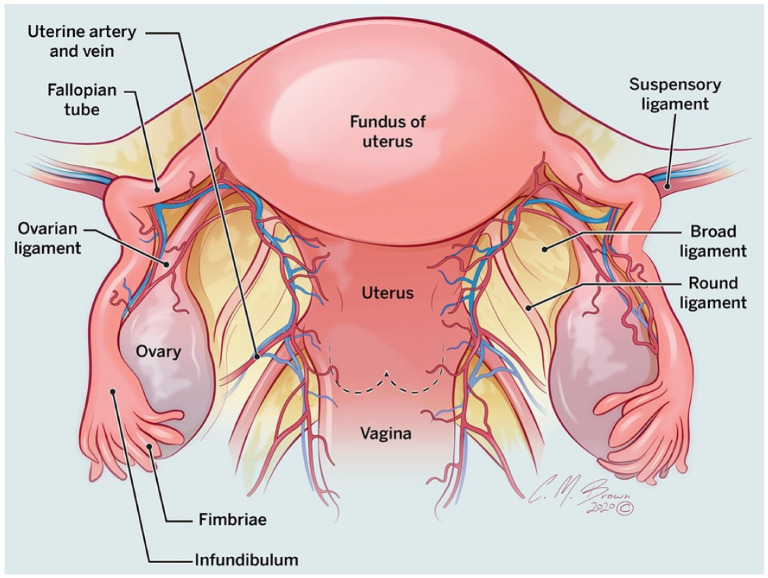
Coronal drawing showing the anatomy of the uterus and ovary.

Localization of pelvic masses can be challenging, notably when anatomic landmarks are obscured or distorted. Identification of the ovaries is an initial step in discerning an intraovarian or extra-ovarian location. Each ovary is suspended in the pelvic peritoneal cavity by three structures: the mesovarium, anchoring the ovary to the posterior surface of the broad ligament; the ovarian ligament, anchoring the ovary to the uterus; and the suspensory ligament, anchoring the ovary to the pelvic sidewall (Figure 1) [5]. Both the ovarian and the suspensory ligaments have different degrees of laxity and, therefore, the ovaries are mobile with variable position and orientation, especially in postpartum women. To locate the ovaries, identify the ovarian veins from their superior drainage confluence—the left ovarian vein drains into the left renal vein and the right into the inferior vena cava. Follow these veins caudally anterior to the psoas muscle and into the pelvis, where they join with the suspensory ligaments. Then, track the suspensory ligament medially to ultimately reach the ovaries. The pelvic ureters are a useful guide since they will be located posterior or posterolateral to the ovary [5].

**Figure 2:**
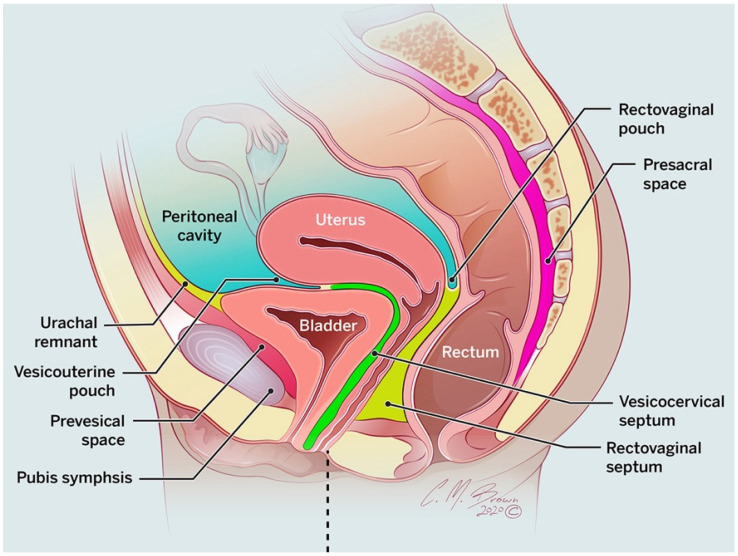
Sagittal drawing showing the inferior boundaries of the peritoneal cavity. Recesses of peritoneal reflections are formed at the vesicouterine pouch and rectovaginal pouch. Inferior to these pouches are fatty septa forming the vesicocervical and rectovaginal septa, respectively.

The pelvis is divided into intraperitoneal and extraperitoneal spaces. The peritoneum is a thin membrane that divides the pelvis into these two spaces. The pelvic peritoneum forms this partition as it drapes over the surfaces of the bladder dome, uterus, and anterior upper third of the rectum [6]. In females, the peritoneum is discontinuous at the ostia of the oviducts and provides a potential route of communication between the two pelvic compartments [6]. Reflections of the peritoneum form potential spaces in females. Anteriorly, the vesicouterine space is between the bladder and the uterus; posteriorly, the rectouterine pouch of Douglas is between the rectum and uterus/upper posterior vagina (Figure 2). Notably, the rectouterine pouch is the most dependent space of the peritoneal cavity since it is the most posterior and caudal in location. The lateral spaces are known as the paravescial recesses [6]. Altogether, the pelvic intraperitoneal space is located centrally in the pelvis and contains the ovaries, small intestine, colon, and upper one-third of the rectum.

**Figure 3:**
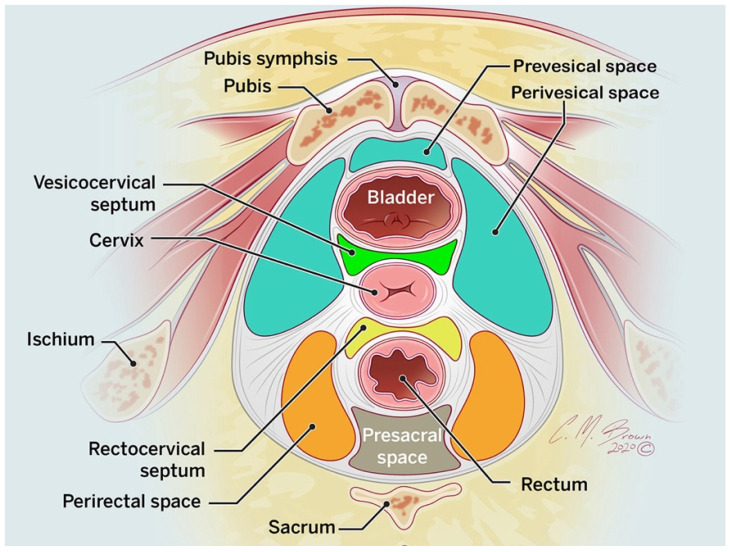
Axial drawing of the low pelvis, illustrating the division of the pelvic extraperitoneal compartments into prevesical and perivesical spaces anteriorly and the perirectal and presacral spaces posteriorly.

The pelvic extraperitoneal space surrounds the intraperitoneal space. This space contains the bladder, uterus, pelvic ureters, lower two-thirds of the rectum, iliac vessels, pelvic lymph nodes, and pelvic sidewall. The pelvic extraperitoneal space can be divided into the anterior, middle, and posterior compartments. The anterior compartment contains the prevesical (retropubic space of Retzius) and perivesical spaces. The prevesical space is a potential space anterior to the urinary bladder, between the infraumbilical abdominal wall and the umbilicovesical fascia. The perivesical space surrounds the bladder. The middle compartment contains the vagina, cervix, and uterus, whereas the posterior compartment contains the perirectal (mesorectum) and presacral (retrorectal) spaces (Figure 3) [7].

## 3. Is the Mass Ovarian or Extra-ovarian in Origin?

Identification of a normal ipsilateral ovary, as described above, excludes ovarian origin of a mass. In cases of masses abutting the ovary, multiple imaging signs help to assess the origin of the mass. Signs suggestive of ovarian origin include a “beak” sign, which is a sharp angle between the ovary and the mass, forming a beak shape at the edge of the ovary; the “ovarian vascular pedicle” sign, which is a depiction of a direct joining of the normal or asymmetrically enlarged gonadal vein to the mass; the “phantom organ sign”, which is an undetectable ovary provided that there is no history of surgical resection or transposition; and the “embedded organ sign”, which is when the ovary appears embedded in the mass [3,8,9]. On the other hand, there are signs suggestive of extra-ovarian origin, such as the “claw sign” and the “bridging vessel” sign, that indicate a uterine origin of the mass, which will be described in detail later, following the paraovarian cyst (Section 5.1.5) and leiomyoma (Section 5.2.2) sections, respectively. Fallopian tube disease is usually characterized from ovarian masses with MRI by identifying the tubular nature of the structure [3].

The direction of organ displacement can often be a clue to the origin of a pelvic mass. Intraperitoneal masses will result in lateral or posterior displacement of the uterus, rectosigmoid colon, and iliac vessels. Masses in the rectouterine pouch of Douglas will result in anterior displacement of the uterus and posterior displacement of the rectum. In contrast, extraperitoneal masses can result in anterior or central displacement of the uterus, rectosigmoid colon, and iliac vessels. Extraperitoneal masses can also efface the pelvic sidewall muscles. Prevesical masses can displace the bladder posteriorly [3]. Accurate localization of the pelvic mass as an extraperitoneal lesion excludes the ovarian origin of the mass.

## 4. Imaging

Ultrasound (US) imaging is considered a first-line modality to image female patients with pelvic symptoms. US is relatively inexpensive and easily accessible. However, it is limited in assessing the extent of disease and is operator dependent. Computed tomography (CT) is increasingly used in emergency and ambulatory settings, so it may be the initial modality for many patients. Limitations of CT include its use of ionizing radiation and a relatively low contrast resolution. Magnetic resonance imaging (MRI) reigns supreme in characterizing pelvic lesions secondary to excellent anatomic depiction and soft-tissue contrast. Sequences like diffusion-weighted imaging (DWI) and dynamic contrast enhancement bolster its capabilities in lesion evaluation [1,10]. Both contrast-enhanced MRI and ultrasound have shown high sensitivity of 96% and 100%, respectively, in assessing adnexal mass, but MRI had much higher specificity and accuracy of 85–98% and 90–99%, respectively, compared to 45–95% and 65–72%, respectively, in ultrasound [11].

Quantitative dynamic contrast-enhanced magnetic resonance imaging (DCE-MRI) is a valuable complementary tool in the characterization of complex adnexal mass. The values of relative signal intensity and the volume transfer coefficient (Ktrans) can differentiate benign adnexal masses from ovarian malignancy with accuracy, sensitivity, and specificity of 79.6%, 96%, 66.7% and 80%, 86%, 74%, respectively [12]. A type I time–intensity curve (TIC) confirms a benign adnexal lesion with 53% sensitivity and 100% specificity, whereas a type III TIC confirms malignant adnexal lesion with 71% sensitivity and 100% specificity [13]. Molecular imaging techniques are complementary modalities, mainly used in the staging of ovarian cancer and treatment response assessment [14]. PET/CT has a reported sensitivity of 52–58% and specificity of 76–78% in the diagnosis of ovarian cancer and it is not recommended for the characterization of adnexal masses secondary to the high false-positive results with hydrosalpinx, pedunculated fibroid, and endometriosis and false-negative results with borderline ovarian tumors and low-grade and early adenocarcinomas [15].

## 5. Intraperitoneal Lesions

### 5.1. Extra-Ovarian Adnexal Lesions

#### 5.1.1. Hydrosalpinx and Hematosalpinx

The fallopian tubes are located at the superior margin of the broad ligament (Figure 1). The fallopian tubes connect the ovaries to the uterus and function to transport the ovum and clear physiologic fluid into the peritoneal cavity through an opening at the fimbriated infundibulum (Figure 1). If the tube becomes obstructed, fluid accumulates and results in progressive dilatation. The dilated fallopian tube can contain simple fluid (hydrosalpinx), blood (hematosalpinx), or pus (pyosalpinx) and can mimic ovarian cysts and cystic neoplasms.

Hydrosalpinx most often occurs due to adhesions from pelvic inflammatory disease. Hydrosalpinx can be asymptomatic but may result in infertility or pelvic pain [16]. On US, the classic finding of hydrosalpinx is a C- or S-shaped anechoic fluid-filled structure. When the fallopian tubes are imaged in cross-section, hydrosalpinx may demonstrate incomplete protrusions in a “cogwheel” configuration, representing thickened endosalpingeal longitudinal folds. On CT and MRI, hydrosalpinx appears as dilated fallopian tube adjacent to the uterus containing simple fluid with thin, mildly enhancing walls [17]. Atypical appearance of hydrosalpinx as a multiloculated cystic mass with papillary projections may mimic borderline ovarian malignancy in postmenopausal women with atrophic ovaries [4].

Hematosalpinx can occur in the setting of endometriosis, ectopic pregnancy, adnexal torsion, malignancy, or trauma. On US, hematosalpinx appears like hydrosalpinx but will show internal low-level echoes reflecting blood products. On MRI, hematosalpinx demonstrates an abnormal signal on a T1-weighted image (T1WI) and T2-weighted image (T2WI), depending on the age of blood products, and lack of T2 shading can differentiate hematosaplinx from an endometrioma [17].

#### 5.1.2. Pelvic Inflammatory Disease, Pyosalpinx, and Tubo-Ovarian Abscess

Pelvic inflammatory disease (PID) represents an ascending upper genital tract infection originating from the vagina and cervix. PID is a spectrum of disease entities, manifesting as salpingitis, pyosalpinx, or tubo-ovarian abscess (TOA). PID is often secondary to an ascending infection from the vagina and cervix and commonly affects both fallopian tubes. The most common infecting organisms are *Neisseria gonorrhoeae* and *Chlamydia trachomatis,* for which 30–40% of cases have polymicrobial infection, whereas tuberculosis and actinomycosis are much less common causes [17]. Relevant clinical information of sexual history, elevated inflammatory markers, and cervical motion tenderness/mucopurulent discharge on pelvic exam are essential for imaging interpretation [17].

**Figure 4:**
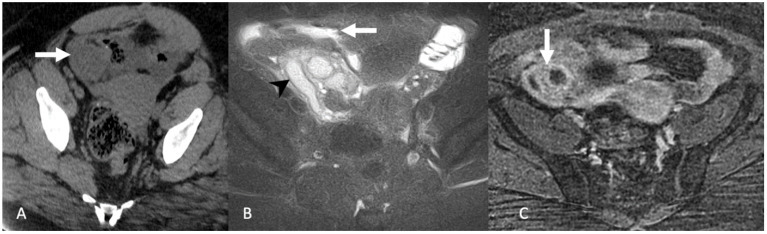
Axial non-contrast-enhanced computed tomography (**A**) demonstrates a rounded soft tissue mass in the right lower quadrant (arrow). Axial T2-weighted magnetic resonance image with fat saturation (**B**) demonstrates a dilated right fallopian tube (arrowhead) with surrounding inflammation/edema (arrow). Axial postcontrast T1-weighted magnetic resonance image (**C**) shows wall enhancement of the fallopian tube (arrow). Findings are consistent with pyosalpinx.

Salpingitis occurs when the ascending infectious process results in a nondilated fallopian tube with wall thickening, hyperenhancement, and adjacent reactive inflammation. Pyosalpinx represents the accumulation of pus due to obstruction at the fimbriated end. On US, pyosalpinx appears as a tubular structure with a thickened wall that is distended with echogenic fluid and debris and possibly a fluid-debris level. On CT, pyosalpinx presents as dilated tube with an enhancing wall (Figure 4) and complex, heterogeneous internal fluid that may have a fluid-debris level. On MRI, pyosalpinx appears as a T2 hyperintense tubular structure with a variable signal on T1WI depending on the fluid content and an enhancing thickened wall without internal enhancement on postcontrast T1WI (Figure 4). Inflammation may extend to the adjacent parametrial fat and adjacent pelvic organs and this helps to distinguish pyosalpinx from hydrosalpinx on MRI [17].

**Figure 5:**
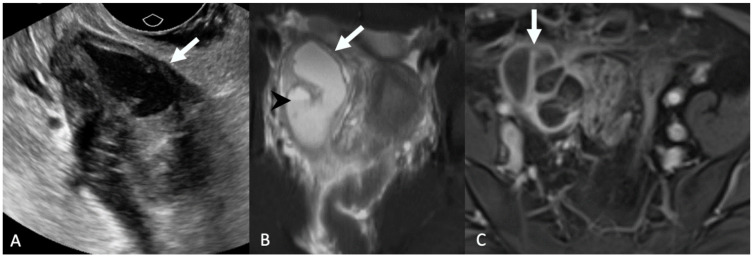
Grayscale transabdominal ultrasound image (**A**) demonstrates a complex right adnexal cystic and solid mass (arrow). Axial T2-weighted magnetic resonance image with fat saturation (**B**) shows the complex cystic right adnexal mass with a fluid-fluid level (arrowhead) and wall thickening (arrow). Axial post-contrast T1-weighted magnetic resonance image (**C**) demonstrates multiple enhancing septations corresponding to the TOA (arrow).

TOA represents the continuation of the ascending infectious and inflammatory processes in 20% of PID cases [11]. The resulting adnexal mass engulfs the fallopian tube and ovary and distorts anatomic boundaries. The TOA may be mistaken for ovarian cancer when the clinical picture does not align with infection [4]. On imaging, TOA appears as a complex solid and cystic mass with wall thickening and uniform septations with mural irregularity and possibly fluid-debris levels. Although internal gas is rarely present [8], its presence is highly specific for TOA [17]. The abscess will demonstrate restriction on DWI. Hemorrhagic and proteinaceous components may be present within the adnexal complex. When present, these components will appear as a high signal on T1WI [18]. A thin rim of hyperintense signal along the inner wall on T1WI may represent granulation tissue [19]. The internal components and surrounding parametrial edema will demonstrate heterogeneously high signal on T2WI. The abscess walls, septa, and surrounding inflammatory stranding will enhance on postgadolinium administration, the latter of which is attributed to fibrosis and adhesions (Figure 5) [19].

#### 5.1.3. Adnexal Ectopic Pregnancy

**Figure 6:**
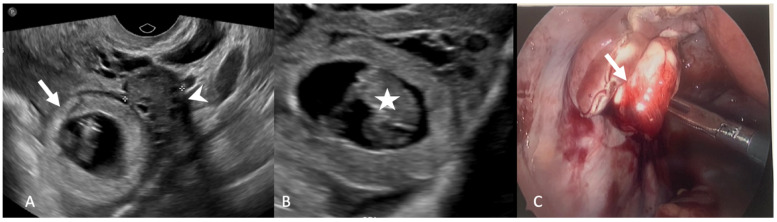
Grayscale transvaginal ultrasound images (**A**,**B**) of the left adnexa show a large gestational sac (arrow) adjacent to the ovary (arrowhead) with a fetal pole (star). Intraoperative laparoscopic image (**C**) demonstrates a ruptured left tubal ectopic pregnancy (arrow).

Ectopic pregnancy is a significant cause of maternal mortality in the first trimester [20]. The most common site of ectopic implant is in the fallopian tubes. Patients often present acutely, and US is used as the primary imaging modality. On US, the tubal ectopic pregnancy can appear as a complex adnexal mass or gestational sac that is separate from the ovary (Figure 6) and may have an echogenic peripheral ring when unruptured or peripheral “ring of fire” denoting hypervascularity on color Doppler. MRI can be utilized when US findings are indeterminate. A ruptured ectopic pregnancy can form a pelvic hematoma, discussed later under Section 7.1 [21,22,23]. Chronic ectopic pregnancy can mimic ovarian malignancy and it represents 1.7% of pelvic masses mimicking ovarian tumors [1].

#### 5.1.4. Adnexal Torsion

Although this article’s focus remains limited to extra-ovarian pathologies, torsion is included because of its mechanism of twisting along the long axis of the fallopian tube and vascular pedicle. Adnexal torsion is twisting of the ovary, and often the fallopian tube, on its ligamental supports, causing vascular compromise [24]. It occurs most commonly in premenopausal patients who present with colicky lower abdominal pain radiating to the groin and adnexal tenderness on palpation. Predisposing factors include polycystic ovaries, long ovarian ligaments, pregnancy, and ovarian hyperstimulation syndrome related to in vitro fertilization (IVF). Adnexal torsion is usually associated with an ovarian cyst or benign ovarian neoplasm in most cases [16,17,25].

**Figure 7:**
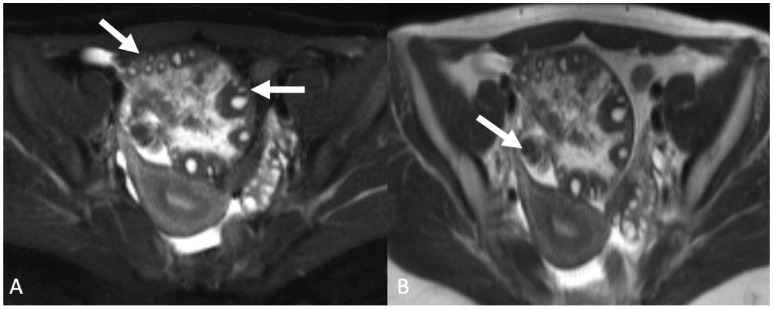
Axial T2-weighted magnetic resonance image with fat saturation (**A**) demonstrates an enlarged, edematous ovary with peripheralized follicles (arrows). Axial T1-weighted magnetic resonance image without fat saturation (**B**) shows twisting about the vascular pedicle (arrow). Findings are consistent with adnexal/ovarian torsion.

At first, venous flow is compromised and results in vascular congestion and edema. If left untreated, the arterial flow becomes compromised and leads to infarction and necrosis. On grayscale US, the ovary can vary from normal to stromal engorgement with peripheralized follicles [26]. There may be variable blood flow on color Doppler, depending on the stage of torsion [27]. On CT and MRI, the ovary can become enlarged and medially displaced towards the center of the pelvis and twisted pedicle whirlpool or thickening of the pedicle can be discerned in 31–100% and 17–97% of the cases [24]. On MRI, the ovary appears enlarged and edematous with high-signal peripheralized follicles on T2WI (Figure 7) and the fallopian tube appears twisted with wall thickening [24,28]. MRI can detect even subtle twists, given the superior spatial resolution. Infarction within the ovary or the fallopian tube appears on CT with intrinsic hyperattenuating areas and on MRI as increased signal on fat-suppressed T1WI and restricted diffusion on DWI, indicative of hemorrhage. The absence of enhancing components following contrast is diagnostic of vascular compromise [24,25].

#### 5.1.5. Paraovarian Cyst

**Figure 8:**
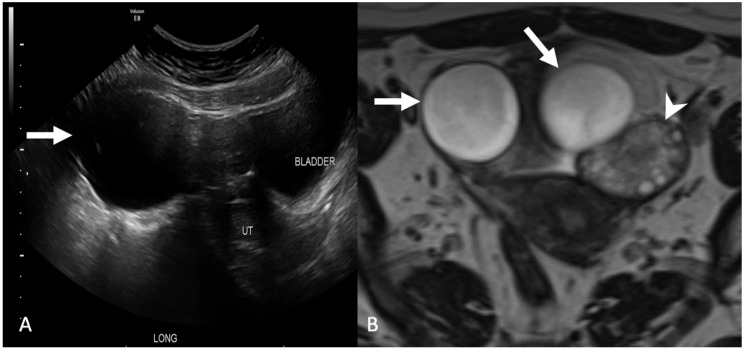
Grayscale transabdominal ultrasound image (**A**) shows a large adnexal cyst (arrow). Axial T2-weighted magnetic resonance image (**B**) demonstrates two cysts in the adnexa (arrows) separate from the ovary (arrowhead). Findings are consistent with a paraovarian cyst.

A paraovarian cyst is made up of the congenital remnants of the embryonic Wolffian duct, originating from the mesosalpinx at the upper border of the broad ligament. These cysts represent 10–20% of all adnexal masses [29]. Paraovarian cysts are often simple and rarely complicated by hemorrhage, debris torsion, and malignant transformation. Identifying the location of a cyst as ovarian or paraovarian can be challenging; gentle pressure with a transvaginal probe can be applied to separate the paraovarian cyst from the adjacent ovary. Additionally, paraovarian cysts lack the crescentic ovarian parenchyma at the periphery “claw sign” that can be seen with eccentric ovarian cysts [29,30]. MRI help identify the paraovarian cysts separate from the ovary and the uncomplicated paraovarian cysts usually show hypointense T1 signals and hyperintense T2 signals, similar to functional cysts (Figure 8) [1].

#### 5.1.6. Peritoneal Inclusion Cyst

**Figure 9:**
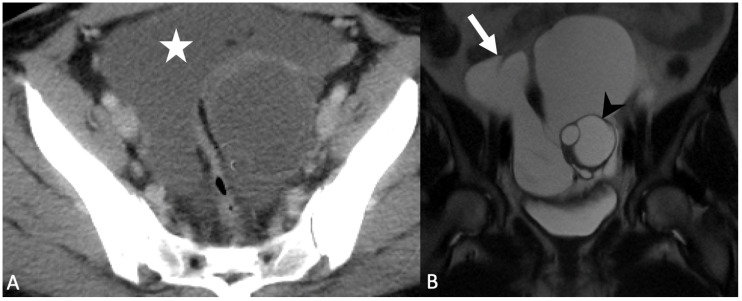
Axial contrast-enhanced computed tomography image (**A**) demonstrates an ill-defined pelvic cyst (star) with poor visualization of the ovaries. Coronal T2-weighted magnetic resonance image without fat saturation (**B**) shows a large cystic collection with septations (arrow) surrounding the left ovary containing follicles (arrowhead). Findings are consistent with a peritoneal inclusion cyst.

A peritoneal inclusion cyst occurs when benign serous ovarian fluid accumulates in entrapped reactive mesothelium, particularly following an infectious or inflammatory process (i.e., surgery, endometriosis, or PID) [31]. These cysts can be simple or multiloculated and will conform to the contour of the adjacent structures. The ovary can become enveloped (but not penetrated) by the cyst and may become suspended in a “spider web” configuration [32]. On US, peritoneal inclusion cysts appear as an anechoic cystic structure. Color Doppler US may demonstrate mildly thickened internal septations containing low-resistance septal vascularity. The cyst conforming to the peritoneal space is often better appreciated on CT or MRI and the entrapped normal-sized ovary with follicles within cystic locules is often characteristic of the peritoneal inclusion cyst (Figure 9). However, the ovary, when not containing follicles, can be mistaken for a solid component in a multilocular cystic mass, mimicking ovarian neoplasm [4]. Peritoneal inclusion cysts do not have malignant potential [31]. Hormonal therapy can be used to reduce the production of serous ovarian fluid in patients with persistent pain [33].

### 5.2. Uterine Lesions

#### 5.2.1. Uterine Vasculature

Dilated uterine vasculature or varices occur in patients with an upstream venous obstruction or incompetent valves. When large, these vessels can mimic a cystic ovarian mass. On US with color Doppler, the mass demonstrates distinct vascularity. On contrast-enhanced CT and MRI, the dilated uterine vasculature will enhance similarly to the adjacent iliac vessels. Pulsation artifacts and flow voids can be present on MRI. Accurately identifying this entity on imaging will obviate a biopsy and can prevent potentially catastrophic large-volume pelvic hemorrhage [34].

#### 5.2.2. Leiomyoma

Leiomyoma, also known as fibroid, is a common benign hormone-responsive tumor. This well-circumscribed tumor contains whorled smooth muscle and connective fibrous stroma. Although submucosal and intramural fibroids rarely present as a diagnostic dilemma, pedunculated or parasitized fibroids can sometimes be less noticeable. Pedunculated fibroids can show a connection with the myometrium [1,35]. Parasitized fibroids are extra-uterine fibroids that receive vascularity entirely from neighboring adnexal structures. These parasitized fibroids are thought to represent pedunculated fibroids that completely detach from the uterus [35]. Round and broad ligament fibroids can be considered parasitized fibroids [36,37].

**Figure 10:**
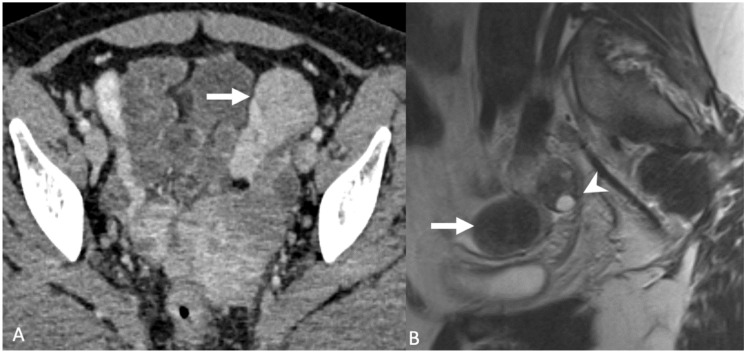
Axial contrast-enhanced computed tomography image (**A**) shows an enhancing mass in the region of the left round ligament (arrow). Sagittal T2-weighted magnetic resonance image (**B**) shows a T2 hypointense mass (arrow) separate from the left ovary (arrowhead). Findings are consistent with a round ligament fibroid.

Pedunculated and parasitized fibroids have similar imaging features of common uterine fibroids with well-defined borders and whorled internal components. On US, pedunculated fibroids contain a stalk connecting the fibroid with the uterus. This finding is best seen on color Doppler with a “bridging vessel” sign representing vessels connecting the mass with the uterus. Multiplanar reconstructions on CT or MRI can assist with anatomic detail. MRI helps characterize internal hemorrhagic (high signal on T1WI), fatty (low signal on fat-saturated sequences), or cystic (non-enhancing, high signal on T2WI) components. MRI also helps in doubtful cases through detection of normal ovaries separate from these pelvic masses (Figure 10) [7].

### 5.3. Peritoneal Disease

#### 5.3.1. Disseminated Peritoneal Leiomyomatosis

**Figure 11:**
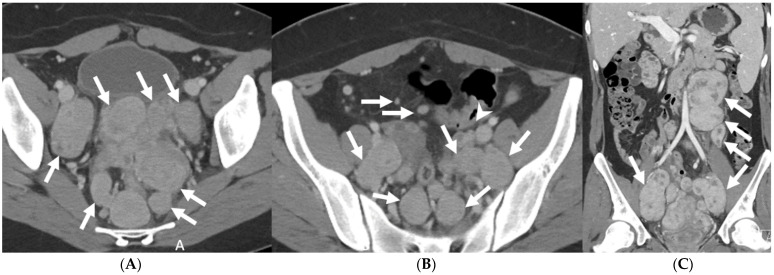
Axial (**A**,**B**) and coronal (**C**) contrast-enhanced computed tomography images show multiple enhancing, smoothly marginated masses (arrows) throughout the abdomen and pelvis, most consistent with disseminated leiomyomatosis.

Disseminated peritoneal leiomyomatosis, also known as leiomyomatosis peritonealis disseminata, is a benign hormone-responsive process often found incidentally among premenopausal women [6]. These solid intraperitoneal masses are composed of smooth muscle. While the larger masses mirror the imaging characteristics of uterine leiomyomas, smaller lesions can be more challenging to characterize. On CT, disseminated peritoneal leiomyomatosis presents as multiple solid and complex well-circumscribed soft tissue masses that mimic a leiomyomatous uterus, with either a postcontrast enhancement similar to the normal uterine parenchyma or possibly a heterogeneous enhancement pattern (Figure 11) [38]. On MRI, these smooth muscle masses are well-circumscribed with hypointense signal on T2WI and isointense to muscle on T1WI. In contrast to peritoneal carcinomatosis, disseminated peritoneal leiomyomatosis is well-circumscribed and not associated with omental involvement, ascites, or lymphadenopathy [38]. Given that splenosis from prior trauma can mimic this entity, interrogation of the left upper quadrant is necessary [39].

#### 5.3.2. Peritoneal Carcinomatosis

Peritoneal carcinomatosis occurs due to metastatic malignant cells seeding the peritoneal cavity. Notably, peritoneal carcinomatosis can occur in the setting of metastatic ovarian cancer. Initially, small, irregularly spiculated soft tissue nodules stud along the peritoneal surface. On CT, peritoneal implants typically demonstrate soft tissue density. However, mucinous peritoneal deposits can demonstrate low attenuation. MRI has an improved detection sensitivity due to the superior soft tissue contrast. Specifically, DWI and postcontrast sequences are beneficial in identification [3,40]. Ascites from proteinaceous exudate is often present and appears anechoic on US. As the disease progresses, the soft tissue can infiltrate or encase the abdominal viscera [6].

#### 5.3.3. Peritoneal Tuberculosis

Peritoneal tuberculosis represents 37.2% of pelvic masses mimicking ovarian tumors [2] and occurs from the reactivation of latent *Mycobacterium tuberculosis* bacteria by hematogenous seeding of the peritoneum from a pulmonary source [41]. Less frequently, peritoneal tuberculosis can occur in active tuberculosis by hematogenous seeding or direct infection from the small bowel or fallopian tubes [42]. The vast majority of patients with peritoneal tuberculosis have ascites with proteinaceous exudate. Imaging features of peritoneal tuberculosis can overlap with peritoneal carcinomatosis. On US, ascites may be anechoic or hypoechoic. On CT, the ascites can be accompanied by peritoneal nodularity or soft tissue masses with central low attenuation components representing the tubers [43].

### 5.4. Gastrointestinal Lesions

#### 5.4.1. Appendicitis

Inflammation of the appendix (appendicitis) in the right lower quadrant can abut or involve the right adnexa, particularly with disease progression and abscess formation. Inflammation of diverticula from the cecum or sigmoid colon (diverticulitis) can also present similarly. Acute appendicitis presents with nausea and vomiting, accompanied by poorly defined periumbilical pain localized to the right lower quadrant. The inflamed blind-ending tubular appendix will be dilated with wall thickening. Transabdominal US of the right lower quadrant with graded compression can help identify appendicitis, but this is operator dependent and limited in patients with a large body habitus [44]. When the dilated appendix is seen, it can be accompanied by surrounding echogenic fat, representing inflammation. On CT and MRI, appendicitis demonstrates edematous wall thickening with edema and inflammation extending to the periappendiceal fat [25].

#### 5.4.2. Appendiceal Mucocele and Pseudomyxoma Peritonei

Appendiceal mucocele represents chronic cystic dilatation of a mucin-filled appendix. Mucocele is histologically classified as a simple mucocele (accumulation due to appendicolith, tumor, or scarring from prior appendicitis), focal or diffuse mucosal hyperplasia, mucin cystadenoma (approximately 50% of cases), and cystadenocarcinoma [4,29,45]. Appendiceal mucocele typically occurs in middle-aged patients. Complications include superimposed infection (appendicitis), adjacent ureteral obstruction, torsion, ileocolic intussusception, and rupture. Since mucocele can increase the risk of neoplasms involving the colon and ovaries [45], these structures should be closely evaluated. Cross-sectional imaging can help correctly identify the appendiceal origin of the cystic mass to avoid misdiagnosis of an ovarian neoplasm [4]. On US, uncomplicated appendiceal mucocele appears as a cystic tubular structure originating from the cecum, sometimes with low-level echoes. The finding of a concentric, echogenic “onion skin sign” is a specific finding of appendiceal mucocele [46]. On CT, the cystic appendiceal mass is homogeneously cystic with simple fluid attenuation. Calcifications on US or CT can be seen in about half of the cases [35]. On MRI, the appendiceal mass demonstrates an increased signal on T2WI. Wall thickening and hyperenhancement are imaging features suggestive of superinfection, while an enhancing soft tissue nodule suggests malignancy [4,29].

**Figure 12:**
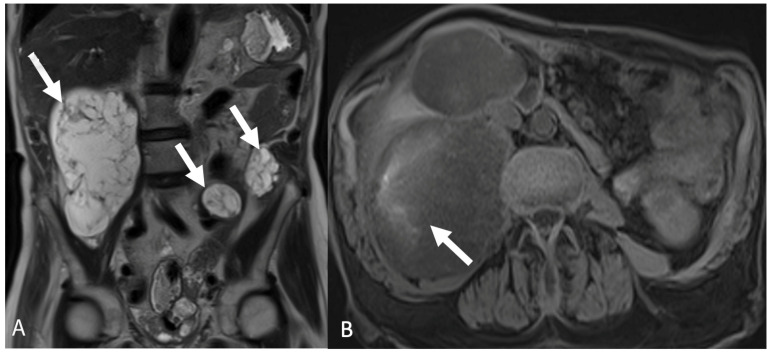
Coronal T2-weighted magnetic resonance image (**A**) shows multiple hyperintense cystic lesions with thin septations in the abdomen (arrows). Axial precontrast T1-weighted magnetic resonance image with fat saturation (**B**) shows mild intrinsic hyperintense signal within the peritoneal masses secondary to mucinous material (arrow). Findings are consistent with pseudomyxoma peritonei.

Appendiceal cystadenoma and cystadenocarcinoma have a greater propensity to rupture due to their relatively larger size [45]. Following penetration or rupture, mucous material seeds within the peritoneal cavity. This extraluminal mucus, called pseudomyxoma peritonei, can involve the ovaries, mimicking a primary ovarian malignancy. On US, pseudomyxoma peritonei contains non-mobile echogenic foci and septations due to the mucinous component. On CT, the hypoattenuating mucinous material coats the peritoneal surfaces, creating a scalloping appearance along the visceral surfaces. On MRI, this mucinous material exhibits a high signal on T2WI and intermediate-to-high signal on T1-WI (Figure 12). The bowel is displaced centrally and posteriorly instead of the floating appearance with simple ascites [10,47]. The mass effect can obstruct the bowel, ureters, and venous structures. Most deaths from pseudomyxoma peritonei are due to intestinal obstruction [48]. Treatment for pseudomyxoma peritonei is surgical debulking and intraperitoneal chemotherapy [45].

#### 5.4.3. Gastrointestinal Stromal Tumor (GIST)

**Figure 13:**
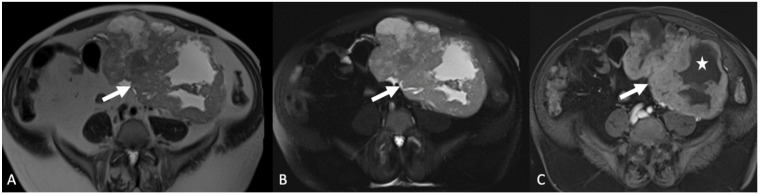
Axial T2-weighted magnetic resonance images without (**A**) and with (**B**) fat saturation show a heterogenous mass in the left lower quadrant (arrows) with a central cystic component. On sequential images, the mass could be seen originating from the small bowel. Axial postcontrast T1-weighted magnetic resonance image (**C**) shows heterogenous enhancement with central cystic necrosis (star). The operative report described the mass as separate from the left ovary (image not shown) and the pathology was consistent with a gastrointestinal stromal tumor (GIST).

GIST is the most common mesenchymal tumor of the gastrointestinal tract and represents approximately 20% of soft tissue sarcomas [49]. GIST has a peak incidence at 50 to 60 years of age, with a slight male predominance. GIST expresses the KIT stem cell factor receptor protein, conferring resistance to apoptosis and unchecked cell growth. GIST usually arises from the stomach (70%), and small intestine (25%), and less commonly arises from the esophagus, colon, and rectum [50]. On MRI, larger GISTs can demonstrate internal cystic necrosis or degeneration, with high-signal regions on T2WI and heterogeneous enhancement on postcontrast T1WI (Figure 13). Smaller GISTs contain more uniform signal intensity and enhance homogeneously. Nearly 20–30% of GISTs are malignant at diagnosis, with the peritoneum being the most common site for metastases. Large size, internal cystic changes, and low signals on apparent diffusion coefficient (ADC) imaging suggest higher malignant potential [51]. Rectal GIST often overlaps with other rectal neoplasms, though the invasion of surrounding structures, bowel obstruction, and local lymph adenopathy are uncommon with rectal GISTs. GISTs are treated surgically or with a tyrosine kinase inhibitor [51,52].

## 6. Extraperitoneal Lesions

Anatomic localization of extraperitoneal lesions is essential as these lesions are not seen on laparoscopy and require a posterior approach. Knowledge of the anatomy and pelvic extraperitoneal spaces can narrow the differential diagnosis [3]. Please refer to the anatomy section, Section 2, for a detailed review.

### 6.1. Lymphoma

Clinical features of lymphoma include fever, night sweats, and weight loss. On imaging, lymphoma appears as multi-station adenopathy and splenomegaly. Occasionally, bulky pelvic masses can appear as an ovarian mass. On CT, lymphoma usually appears as mild to moderately enhancing homogenous masses. Lymphomatous adenopathy tends to encase vessels without compression. On MRI, lymphoma appears isointense in T1WI, iso- or hyperintense on T2WI, and moderately enhancing on postcontrast T1WI [53,54,55].

### 6.2. Retrorectal Cysts

Retrorectal cysts are part of a general category of lesions occupying the perirectal and presacral spaces. Lesions in these spaces are commonly classified in the literature based on etiology (e.g., congenital, neurogenic, inflammatory, etc.) [56]. These lesions can also be divided based on their origins from the sacrum, retrorectal space, or rectum [57]. Cystic lesions that arise from the sacrum include spinal meningeal cysts and neurogenic tumors (discussed separately in Section 6.4). Cystic lesions arising from the perirectal or presacral spaces include epidermoid cysts, dermoid cysts, and lymphangiomas. Cystic lesions arising from the rectum, known as enteric cysts, include tailgut cysts and rectal duplication cysts. Accurately classifying the site of origin can help determine the appropriate surgical team and approach [58].

In general, these cystic structures can be unilocular or multilocular. Most lack soft tissue components or internal enhancement [59], but can be complicated by superimposed infection, fistulous connection, hemorrhage, or malignant degeneration. US and CT are beneficial in identifying the cystic lesion, though internal components and anatomic relationships are better visualized with MRI [60].

**Figure 14:**
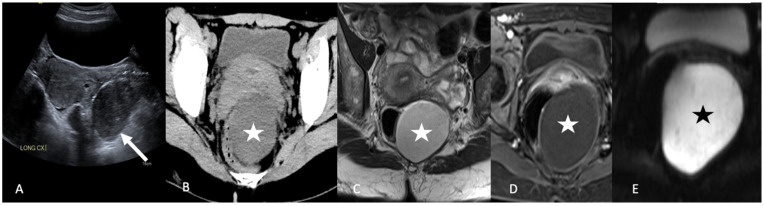
Grayscale transabdominal ultrasound image (**A**) shows a heterogeneous hypoechoic lesion (arrow) posterior to the uterus. Axial contrast-enhanced computed tomography image (**B**) shows the lesion to be cystic and extraperitoneal in location with mass effect on the rectum. Axial T2-weighted magnetic resonance image (**C**) shows a hyperintense extraperitoneal cystic lesion (star). Axial postcontrast T1-weighted magnetic resonance image (**D**) shows no internal enhancement or soft tissue component (star). Axial diffusion-weighted imaging b1000 (**E**) shows marked hyperintensity of the cyst (star). Findings are consistent with an epidermoid cyst.

Epidermoid cyst is an unilocular cyst containing a clear fluid with high signal on T2WI and characteristic restricted diffusion on DWI (Figure 14). Dermoid cysts are fat-containing cystic structures that are ectodermal in origin and reported in up to 25% of postmenopausal women [11]. On MRI, the macroscopic fat component of a dermoid cyst is hyperintense on T1WI, with corresponding signal drop out on fat-saturated sequences. Lymphangioma is a serpiginous cystic structure with thin septations and can span across multiple anatomic compartments [60].

Tailgut cysts, also known as retrorectal cystic hamartomas, are developmental tumors arising from the ectodermal layer. Tailgut cysts often appear multilocular but can be unilocular [61]. On MRI, these cysts are hyperintense on T2WI and can contain hyperintense mucoid material on T1WI [60]. Thin peripheral enhancement may be present in uncomplicated cysts. These cysts can become superinfected, resulting in thickened and enhancing walls. The presence of internal enhancement raises the suspicion for malignancy. Given that about 13% of tailgut duplication cysts undergo malignant transformation [62], surgical excision is recommended, though some patients will undergo active surveillance [63].

Rectal duplication cysts are relatively rare cysts. Best appreciated on MRI, these cysts communicate with the anorectal lumen and are lined by intestinal mucosa and submucosa. Surgical removal of this lesion is also recommended as approximately one of every five cases has malignant potential [64].

### 6.3. Spinal Meningeal Cysts

Neurogenic cysts arise from the nerve root or spinal column and may be confused with cystic ovarian or adnexal mass if there is no apparent neural involvement [65]. Spinal meningeal cysts, also known as perineural or arachnoid cysts, are diverticula arising from the meningeal sac, nerve root sheath, or arachnoid mater. The origin from the nerve root or the thecal sac is best depicted on MRI [29].

Anterior sacral meningocele is a congenital disorder secondary to agenesis of the anterior sacrum, resulting in a scimitar-shaped sacral osseous defect. CT and MRI demonstrate a bony defect of the sacrum with an associated cystic lesion communicating with the thecal sac [66]. Dural ectasia of the lumbosacral spine is caused by the widening of dural sac and neural foramina and occurs in many patients with Marfan syndrome [67]. On imaging, dural ectasia appears as a multilobulated cystic mass extending anteriorly in the pelvis and into an anterior sacral meningocele [4,67].

### 6.4. Neurogenic Tumors

Pelvic extraperitoneal neurogenic tumors can arise from the nerve sheath (schwannoma and neurofibromas), the sympathetic nerve (ganglioneuroma), and the paraganglionic tissues (paraganglioma) [68]. All of these neurogenic tumors may undergo cystic degeneration [61,68].

Extraperitoneal schwannoma is an encapsulated well-defined nerve tumor. On CT, this tumor may contain calcifications. On MRI, schwannoma has low signal on T1WI, high signal on T2WI, and heterogeneous contrast enhancement on postcontrast T1WI. When large, schwannoma can contain regions of cystic necrosis or hemorrhage. Schwannoma has a low risk of recurrence or malignant transformation. Treatment includes surgical resection for symptomatic patients [69].

Neurofibroma is a benign unencapsulated nerve sheath tumor that arises along the course of the nerve [60,70]. Neurofibroma can be multifocal in neurofibromatosis type 1 disease. When located at the neural foramen, neurofibroma appears as a dumbbell shape with osseous expansion, whereas plexiform neurofibroma appears as an extensive infiltrative tumor. On CT, neurofibroma is homogeneously hypoattenuating due to the presence of fat from Schwann cells, adipocytes, and entrapment of adjacent fat. Homogeneous or target-like enhancement can be seen on postcontrast CT images. On MRI, neurofibroma presents with a central hyperintense signal on T1WI due to the neural components. The classic target sign appearance is appreciated on T2WI with a hyperintense peripheral rim and central hypointense component [60,68]. Neurofibroma may degenerate to a malignant peripheral nerve sheath tumor, particularly in the setting of neurofibromatosis [56].

Ganglioneuromas arise from the sympathetic ganglia. On CT, ganglioneuromas appear as a well-defined soft tissue masses with occasional punctate calcifications. On MRI, they are hypointense on T1WI with a “whorled” appearance on postgadolinium images secondary to internal collagen and Schwan cell components [70].

Paraganglioma, also referred to as extra-adrenal pheochromocytoma, contains neural crest elements. Nearly 40% of paragangliomas can produce clinically significant catecholamines, resulting in elevated biochemical markers, including urine metanephrine and vanillylmandelic acid. On CT imaging, paraganglioma appears as a well-defined soft tissue mass that avidly enhances following contrast administration. On MRI, paraganglioma may contain signal voids on T1WI. The classic description of a “light bulb” bright hyperintensity on T2WI is not often seen due to hemorrhage [71]. Surgical resection is the mainstay of treatment, while alpha-blockers are used preoperatively to prevent the sudden release of catecholamines [72].

## 7. Either Intraperitoneal or Extraperitoneal Lesions

### 7.1. Hematoma

**Figure 15:**
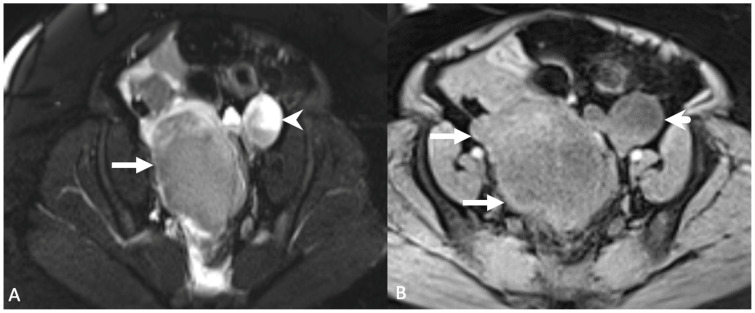
Axial T2-weighted magnetic resonance image with fat saturation (**A**) shows a heterogeneous, slightly hyperintense mass (arrow) centered in the pelvis. Additionally, there is a ruptured left ovarian hemorrhagic cyst with a fluid-fluid level (arrowhead). Axial precontrast T1-weighted magnetic resonance image with fat saturation (**B**) shows hyperintense regions within the collection (arrow). The fluid-fluid level in the left ovarian hemorrhagic cyst is again seen (arrowhead). The pelvic mass was found to represent a pelvic hematoma.

Hematoma can occur following trauma, surgery, interventional procedures, or aneurysm rupture. Depending on the location, it may be challenging to distinguish the ovary from the pelvic hematoma. Imaging of hematoma varies temporally as blood products break down and are reabsorbed. On US, acute hematoma starts as an echogenic collection, then progressively becomes hypoechoic with septations and thickened walls. On CT, acute hematoma starts as a hyperattenuating collection that becomes cystic with thickened walls in about two weeks. On MRI, acute and subacute hematomas have high signals on fat-suppressed T1WI and do not demonstrate enhancement on postcontrast T1WI (Figure 15). As the hematoma ages, the collection shrinks with the formation of a thin, peripheral, low-signal rim on T1WI and T2WI due to the presence of hemosiderin. A subjacent bright inner rim of increased signal on T1WI may form, known as the “concentric ring” sign. Multiphase contrast-enhanced CT or MRI may be necessary to evaluate for active bleeding. If a hematoma arises de novo, postprocessed subtraction images on MRI or follow-up imaging may be required to assess for an underlying lesion [10,73].

### 7.2. Lymphocele, Seroma, and Urinoma

**Figure 16:**
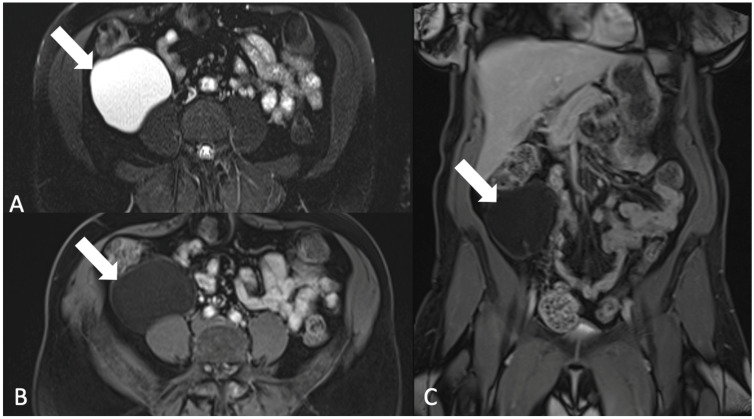
Axial T2-weighted magnetic resonance image with fat saturation (**A**) shows a cyst in the right lower quadrant (arrow). Axial (**B**) and coronal (**C**) postcontrast T1-weighted magnetic resonance images show no internal enhancement. The pathology was consistent with a lymphocele.

Lymphocele is a lymph-containing fluid collection contained within the walls of thin fibrotic tissue. Clinical history is essential as pelvic lymphoceles occur approximately three to eight weeks following lymphadenectomy or renal transplant surgery [29]. Depending on the size and mass effect, a lymphocele may result in lower extremity edema, deep venous thrombosis, or hydroureteronephrosis. Lymphoceles appear as a non-enhancing simple fluid collection along the lymphatic chain (Figure 16). Like other fluid collections, lymphoceles can become complicated by superinfection or hemorrhage [4,10].

Seroma is a fluid collection containing serous fluid that can occur following surgery or trauma. Akin to a lymphocele, seroma can exhibit a mass effect on the adjacent pelvic structures and appears as a non-enhancing simple fluid collection.

Urinoma is a fluid collection that contains extravasated urine. Urinoma can result from urinary tract obstruction (e.g., calculus) or iatrogenic causes, such as following an intervention or surgery. On US, urinoma appears as anechoic simple fluid collection. On CT imaging, urinoma shows fluid attenuation on unenhanced images but may have increased attenuation on excretory or further delayed phase imaging. CT or MR urography can depict the presence and site of extravasation [74].

### 7.3. Pelvic Abscess

Pelvic abscesses represent 17.7% of pelvic masses mimicking ovarian tumors [2] and can form during gynecological processes (i.e., tubo-ovarian abscess), complicated appendicitis, complicated diverticulitis, inflammatory bowel disease, anastomotic leak, or superinfected fluid collections. Clinical history, past surgical history, and relevant laboratory values are necessary for proper interpretation. In general, pelvic abscesses demonstrate thick peripheral wall enhancement, complex internal components containing air (from a fistula or gas-forming organism), and inflammatory changes of the adjacent tissue [4,10].

## 8. Conclusions

Imaging features that mimic ovarian tumors present a diagnostic challenge due to the complex pelvic anatomy and overlapping imaging features of various pelvic lesions. Identifying a normal-appearing ovary is crucial in distinguishing an extra-ovarian pelvic mass from an ovarian tumor. Understanding pelvic anatomy and knowledge of key imaging features can help in anatomic localization and narrowing the differential diagnosis to determine the origin of lesions in the female pelvis. Ultrasound is the primary modality for diagnosis of adnexal lesions and MRI is a problem-solving modality, given it superior soft tissue contrast, guiding optimal patient management.

## Data Availability

Not applicable.
